# A Case of Tracheal Hamartoma Resected with Loop Electrocautery

**DOI:** 10.1155/2013/568590

**Published:** 2013-01-14

**Authors:** Marios Panagiotou, Alexandros Kalkanis, Napoleon Karagiannidis, Vlasis Polychronopoulos

**Affiliations:** ^1^3rd Department of Pulmonology, Sismanoglio General Hospital, 15126 Athens, Greece; ^2^Ygeia Hospital, 15123 Athens, Greece

## Abstract

The authors report on the case of a 67-year-old man with longstanding breathlessness, which was eventually attributed to a fixed mass in the upper third of the trachea causing upper airway obstruction. The lesion was amenable to loop electrocautery resection via flexible bronchoscopy that led to prompt resolution of patient symptoms. Biopsy was consistent with tracheal hamartoma, an exceedingly rare benign tracheal tumor. All the cases of tracheal hamartomas in the literature to date, the application of electrocautery and other methods of interventional bronchoscopy for resection of selected tracheal tumors are discussed.

## 1. Introduction

Primary tracheal tumors are rare in adults and usually malignant (80–90%). Reports regarding the histological distribution of the benign tracheal tumor are scarce, and little information is available about their natural history and behavior. The rarity of cases results in low levels of suspicion among physicians thus leading to substantial delay in correct diagnosis and treatment [1, 2]. 

## 2. Case

A 67-year-old exsmoker man presented with a 5-year history of progressive breathlessness on exertion and no other associated symptoms whatsoever. He had been empirically treated as for chronic obstructive lung disease in the past without any improvement.

**Figure 1:**
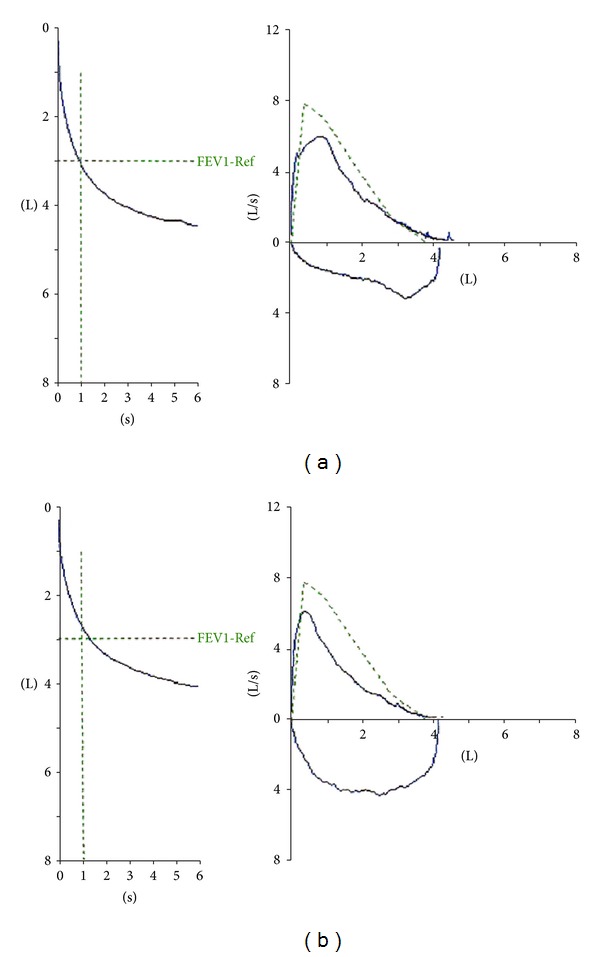
Flow-volume loop before (a) and after (b) the resection of the tracheal tumor.

**Figure 2:**
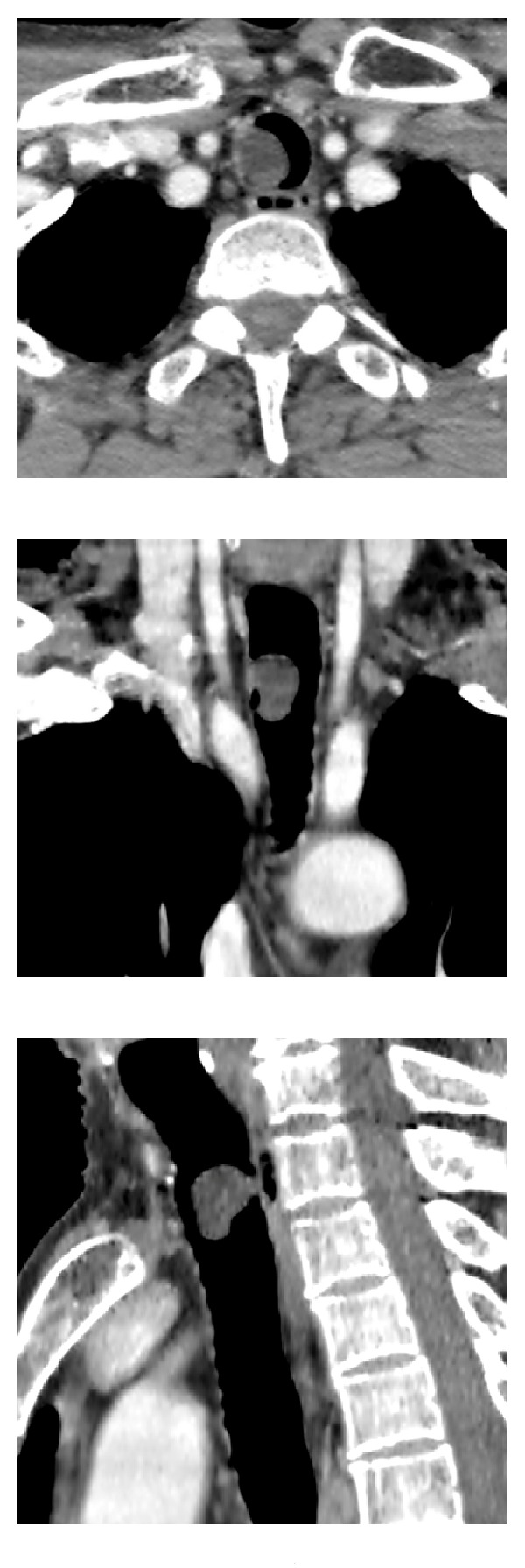
From left to right: axial, coronal, and sagittal CT views of the tumor located in the upper third of the trachea.

Flow-volume loop test revealed a variable extrathoracic airway obstruction (Figure 1). Chest X-ray was normal, but a thoracic CT scan revealed an 18 × 11 × 17 mm polypoid tumor in the upper third of the trachea with fat tissue density and no contrast uptake (Figure 2). The lung fields appeared normal.

**Figure 3:**
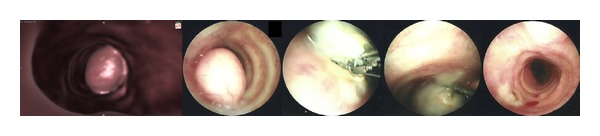
From left to right: CT reconstructed image and bronchoscopic views of the tracheal tumor and its complete resection with the use of loop electrocautery.

**Figure 4:**
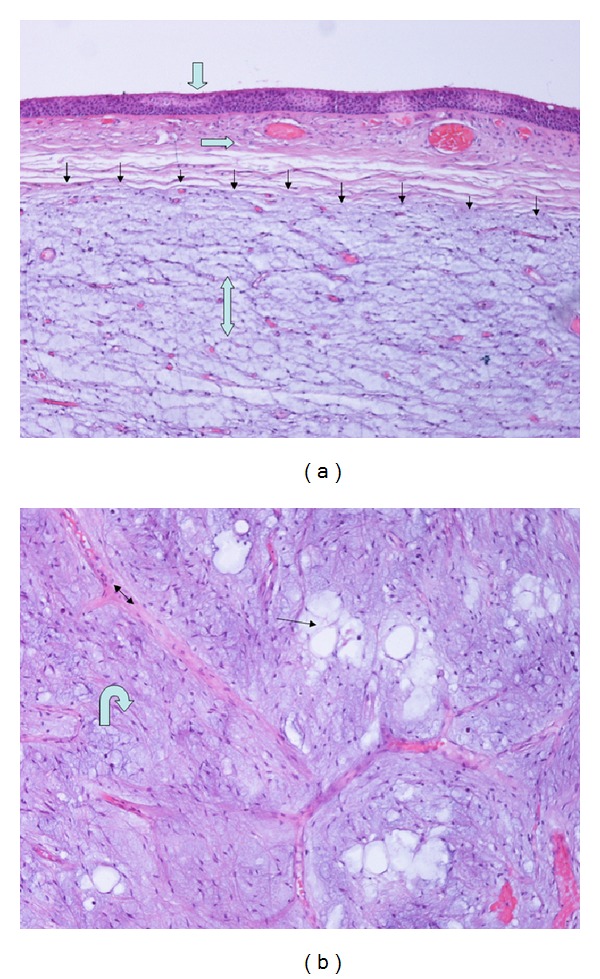
Microscopic features: mucosa with respiratory-type epithelium (fat arrow), vascularized submucosa (horizontal arrow), well-defined hamartoma (thin arrows), myxoid fibrovascular tissue of hamartoma (double arrow) (H-EX100), hondromyxoid lobules (hooked arrow) admixed with adipocytes (thin arrow), and separated by fibrous septa (double arrow) (H-EX400).

The patient underwent flexible bronchoscopy under local anesthesia following an oral approach which demonstrated a white pendulated tumor covered with smooth mucosa attached with a narrow base of 2-3 mm to the right antero-lateral tracheal wall between the second and the third cartilaginous rings form the vocal cords. The lesion was obliterating the tracheal lumen by 80%. An electrocautery loop was passed through the bronchoscope, positioned around the base of the tumor, and tightened to gather the tissue at the point of its attachment to the tracheal wall. The tumor was completely resected by delivering electrocautery. Following resection, the tumor remained attached to the electrocautery loop, and it was removed with the simultaneous withdrawal of the bronchoscope, the electrocautery loop, and the hamartoma en bloc. The procedure was well tolerated, without hemorrhage from the tumor base or other complications, and offered the patient immediate normalization of his breathing and resolution of his stridor (Figure 3). Histological examination of the lesion was diagnostic of hamartoma (Figure 4).

The patient was discharged the next day being completely asymptomatic. A repeat flow-volume loop at three months showed normalization of the inspiratory limb. There had been no recurrence of dyspnea during that period (Figure 2).

## 3. Discussion

Hamartomas are the most common benign tumor of the lung. Grossly, they occur endobronchially (or endotracheally) in about 10% of the cases and intraparenchymally in the remaining 90% [3]. 

Endotracheal hamartomas are excitingly rare amongst other benign tracheal tumors. On reviewing the PubMed database, approximately 20 cases of tracheal hamartoma have been reported from 1959 onwards, including cases in adults and children. One case of each of the followings was found: an endotracheal hamartoma coexistent with an intrapulmonary hamartoma [4], an endobronchial hamartoma accompanied by a similar intrapulmonary lesion [5], and multiple pulmonary hamartomas in trachea, bronchi, and lung parenchyma [6]. Although rare, these cases might seem to support the view that intrapulmonary and tracheobronchial hamartomas are similar, the only difference being the site of origin and the direction of growth [4, 7]. There is one report on an extraluminal tracheal hamartoma presenting as a neck mass in a pediatric patient [8]. With regards to location in the trachea, there seems to be a general agreement that the lower one-half to two-thirds of the trachea is the site of most tumours arising in the adult trachea which makes the present case even more rare. 

Tracheobronchial hamartomas commonly manifest with varying degree of respiratory distress, depending on the degree of luminal obstruction, but manifestations ranging between episodes of pneumonia and acute onset of respiratory failure, cough, hemoptysis, chest pain, and paucity of symptoms have been described [9–11]. Macroscopically, they are often polypoid, either sessile or with a thin pedicle with a tan to pink surface [3]. Chronic inflammation may exist, giving rise to an inflamed surface macroscopically indistinguishable from bronchogenic carcinoma [2]. 

Tracheal tumors are amenable to direct visualization and sufficient sampling by flexible bronchoscopy. Surgical resection has been the treatment of choice in malignant tracheal tumors and benign lesions invading the tracheal wall or attached to the tracheal lumen with a broad base [2]. When definite histological diagnosis of benignity is established, management options include either a conservative watch and wait approach in asymptomatic lesions [4] or, more commonly, resection. Along with surgery, there is now significant growing experience with methods of bronchoscopic resection for central airway tumors such as endobronchial electrocautery, laser, and cryotherapy [9, 11–16]. These methods may serve as the treatment of choice for benign tumors with certain characteristics such as those pendulated and narrow based or for malignant tumors in the elderly or inoperable patients due to lower, though not non-existent [17], risk compared to conventional operative resection. Amongst all, electrocautery represents the most cost-effective technique.

## 4. Conclusion

When added to other published data, this case report of tracheal hamartoma brings the total reported number of this rare localization of a common condition to only around twenty. It also highlights the challenges relating to the timely correct diagnosis of central airway lesions. Based on our experience from the management of this case and on reviewing the relevant literature, we propose that, in the hands of an experienced manipulator, bronchoscopic techniques such as electrocautery may serve as the method of choice for the resection or debulking of selected endotracheal tumors in certain patients; they are safe and straightforward techniques, provide immediate effect, are cost-effective, increase patient satisfaction, and reduce length of hospital stay.

## References

[1] Roby BB, Drehner D, Sidman JD (2011). Pediatric tracheal and endobronchial tumors: an institutional experience. *Archives of Otolaryngology—Head and Neck Surgery*.

[2] Cetinkaya E, Gunluoglu G, Eyhan S (2011). A hamartoma located in the trachea. *Annals of Thoracic and Cardiovascular Surgery*.

[3] Irwin SR, Ernst A, Blackmon S, Fraire EA, Cagle TP, Irwin SR (2010). Pulmonary hamartoma. *Atlas of Neoplastic Pulmonary Disease*.

[4] Suzuki N, Ohno S, Ishii Y, Kitamura S (1994). Peripheral intrapulmonary hamartoma accompanied by a similar endotracheal lesion. *Chest*.

[5] Minasian H (1977). Uncommon pulmonary hamartomas. *Thorax*.

[6] Domínguez H, Hariri J, Pless S (1996). Multiple pulmonary chondrohamartomas in trachea, bronchi and lung parenchyma. Review of the literature. *Respiratory Medicine*.

[7] Bateson EM (1970). Histogenesis of intrapulmonary and endobronchial hamartomas and chondromas (cartilage-containing tumours): a hypothesis. *Journal of Pathology*.

[8] Gross E, Chen MK, Hollabaugh RS, Joyner RE (1996). Tracheal hamartoma: report of a child with a neck mass. *Journal of Pediatric Surgery*.

[9] Altin S, Dalar L, Karasulu L, Çetinkaya E, Timur S, Solmazer N (2007). Resection of giant endobronchial hamartoma by electrocautery and cryotherapy via flexible bronchoscopy. *Tuberkuloz ve Toraks*.

[10] Iusco D, Bobbio A, Donadei E, Carbognani P (2007). Hamartochondroma arising from a tracheal bronchus. *Chirurgia Italiana*.

[11] Hamacher J, Baumann HR, Mordasini C (1993). Diagnosis and treatment of endobronchial and tracheal hamartoma. *Schweizerische Medizinische Wochenschrift*.

[12] Ogawa J, Inoue H, Shohtsu A, Makuuchi H (1991). Tracheal hamartoma—report of a case successfully treated with endoscopic surgery. *Japanese Journal of Surgery*.

[13] Nakano M, Fukuda M, Sasayama K, Nakata T, Fujimoto S, Araki J (1992). Nd-YAG laser treatment for central airway lesions. *Nihon Kyobu Shikkan Gakkai Zasshi*.

[14] Bolliger CT, Sutedja TG, Strausz J, Freitag L (2006). Therapeutic bronchoscopy with immediate effect: laser, electrocautery, argon plasma coagulation and stents. *European Respiratory Journal*.

[15] Sindhwani G, Rawat J, Keserwani V (2012). Role of endobronchial electrocautery in management of neoplastic central airway obstruction: initial experience with seven cases. *The Indian Journal of Chest Diseases & Allied Sciences*.

[16] Rodrigues AJ, Coelho D, Dias Júnior SA, Jacomelli M, Scordamaglio PR, Figueiredo VR (2011). Minimally invasive bronchoscopic resection of benign tumors of the bronchi. *Jornal Brasileiro de Pneumologia*.

[17] Sigurdsson MI, Sigurdsson H, Hreinsson K, Simonardottir L, Gudbjartsson T (2011). Bronchiovenous fistula causing bleeding and air embolism: an unusual complication of bronchoscopic tumor resection. *American Journal of Respiratory and Critical Care Medicine*.

